# Emerging Immune‐Based Therapeutic Strategies in Hepatocellular Carcinoma

**DOI:** 10.1111/liv.70773

**Published:** 2026-07-01

**Authors:** Janine Kah, Werner Dammermann, Stefan Lueth

**Affiliations:** ^1^ Department of Internal Medicine University Medical Center Hamburg‐Eppendorf Hamburg Germany; ^2^ Faculty of Health Sciences Brandenburg Brandenburg Medical School Theodor Fontane Neuruppin Germany; ^3^ Department of Gastroenterology University Hospital Brandenburg Brandenburg an der Havel Germany

## Abstract

Hepatocellular carcinoma (HCC) remains a leading cause of cancer‐related mortality worldwide despite recent advances in systemic therapy. The introduction of immune checkpoint inhibitors has substantially expanded treatment options for advanced disease; however, durable responses are observed only in a subset of patients. Increasing evidence indicates that this limited efficacy is largely driven by the complex immune landscape of HCC, which is shaped by disease aetiology, tumour microenvironment–mediated immune suppression, and impaired innate immune surveillance. Chronic viral hepatitis and metabolically driven liver diseases represent the principal drivers of HCC and profoundly influence immune regulation within the liver. Persistent antigen exposure, metabolic reprogramming, and stromal remodelling contribute to dysfunctional T‐ and natural killer (NK) cell responses, while immunosuppressive components of the tumour microenvironment, including tumour‐associated macrophages, myeloid‐derived suppressor cells, regulatory T cells, and inhibitory ligands such as HLA‐G, facilitate immune escape and therapeutic resistance. In this review, we discuss recent advances in HCC immunotherapy, focusing on emerging checkpoint pathways such as TIGIT and Tim‐3, glypican‐3 targeted cellular therapies and bispecific antibodies, and NK cell‐based therapeutic strategies. We further highlight the role of liquid biopsy approaches for treatment monitoring and biomarker development. Together, these insights emphasize the need for biomarker‐guided patient stratification and integrated therapeutic strategies to improve the clinical efficacy of immunotherapy in HCC.

## Key Points

- The limited efficacy of current immunotherapies in hepatocellular carcinoma (HCC) is strongly influenced by the immunosuppressive tumour microenvironment, including dysfunctional NK‐ and T‐cell responses, tumour‐associated macrophages, myeloid‐derived suppressor cells, regulatory T cells, and inhibitory ligands such as HLA‐G.
- Emerging immune‐based strategies, including TIGIT‐ and Tim‐3‐directed checkpoint inhibition, glypican‐3‐targeted CAR‐T/CAR‐NK cells, bispecific antibodies, and NK cell‐based therapies, show promising potential to overcome immune escape mechanisms in HCC.
- Advances in biomarker‐guided patient stratification, liquid biopsy technologies, and aetiology‐specific immunotherapeutic approaches may improve treatment monitoring and support the development of more effective personalized therapies for HCC.

## Lay Summary

Liver cancer, particularly hepatocellular carcinoma (HCC), is one of the leading causes of cancer‐related deaths worldwide. Although new immunotherapies have improved treatment options, they only work well for some patients. This is because liver tumours develop in a complex immune environment that often suppresses the body’s natural ability to fight cancer. Chronic liver diseases, such as viral hepatitis and fatty liver disease, further weaken immune responses and help tumours evade detection. This review explains how these immune changes influence treatment outcomes and highlights promising new immunotherapies, including therapies that target immune checkpoints, engineered immune cells, and natural killer cells. It also discusses the growing role of blood‐based biomarkers to help identify which patients are most likely to benefit from these treatments, with the goal of advancing more personalized and effective care for liver cancer.

## 1 Global Burden, Aetiology, and Clinical Challenges of HCC

Hepatocellular carcinoma (HCC), the most common malignant liver tumour, is still a major health problem around the world, especially in East Asia and Africa, where it is one of the most common causes of cancer deaths [1, 2]. The dominant risk factors in these regions are chronic infections with hepatitis B (HBV) and hepatitis C viruses (HCV). Globally, approximately 50% of HCC cases are attributable to chronic viral hepatitis, despite widespread implementation of hepatitis B vaccination programs and the availability of curative antiviral therapies for HCV [3].

However, the epidemiological landscape of HCC is evolving. While viral hepatitis continues to drive disease incidence in many parts of the world, incidence and mortality rates are rising rapidly in Western countries [4]. In these regions, metabolic dysfunction‐associated steatotic liver disease (MASLD) and its progressive inflammatory form, metabolic dysfunction‐associated steatohepatitis (MASH), are emerging as major contributors to HCC pathogenesis [5, 6, 7, 8] and are increasingly associated with dysfunctional T‐ and NK‐cell responses [9, 10]. In parallel, landmark pharmacological advances targeting fatty liver diseases are showing promising results. Notably, a large Swedish cohort study recently demonstrated that long‐term use of GLP‐1 receptor agonists, such as semaglutide, is associated with a substantially reduced risk of developing severe liver disease, including cirrhosis and hepatocellular carcinoma, particularly in patients with type 2 diabetes and pre‐existing liver dysfunction [11].

Despite improvements in prevention and risk modification, early detection of HCC remains a major clinical challenge. Surveillance strategies based on ultrasound with or without serum alpha‐fetoprotein (AFP) can improve early diagnosis and survival; however, their implementation remains suboptimal, and most HCC cases are still diagnosed at intermediate or advanced stages [4]. In clinical practice, staging systems such as the Barcelona Clinic Liver Cancer (BCLC) algorithm guide therapeutic decision‐making by stratifying patients according to tumour burden, liver function, and performance status [12]. Nevertheless, even with advances in systemic therapy, recurrence remains frequent, with up to 60% of patients experiencing tumour relapse within five years [13, 14].

These clinical challenges are further compounded by substantial global disparities in HCC care. In many HCC‐endemic low‐ and middle‐income countries, access to immune‐based therapies remains limited due to high drug costs and restricted healthcare infrastructure. Such inequalities are particularly evident in regions such as Central America and the Caribbean, where limited access to diagnostic imaging and systemic therapies continues to impede optimal patient management [15, 16].

### 1.1 Current Advances and Limitations in Systemic Therapy

In recent years, immune checkpoint inhibitors have substantially changed the therapeutic landscape of advanced HCCs. Antibodies targeting the PD‐1/PD‐L1 axis, such as nivolumab, pembrolizumab, and atezolizumab, have demonstrated clinically meaningful therapy outcomes in patients with unresectable HCC [17]. In particular, the combination of the PD‐L1 inhibitor atezolizumab with the anti‐VEGF antibody bevacizumab has become a cornerstone of first‐line systemic therapy following the results of the IMbrave150 trial [18]. Consequently, immune‐based therapies have become increasingly integrated into stage‐specific treatment strategies (Table S1). Immune checkpoint inhibitor combinations are now considered standard first‐line systemic therapies for advanced HCC (BCLC stage C) [18, 19], while ongoing studies are evaluating their combination with locoregional treatments in intermediate disease (BCLC stage B) and as adjuvant strategies following curative therapies in early‐stage disease (BCLC 0/A) [17, 20].

However, updated data from the IMbrave050 trial suggest that the clinical benefit of adjuvant atezolizumab plus bevacizumab in high‐risk HCC may be more limited than initially anticipated, highlighting the need for improved patient stratification and alternative therapeutic strategies [20]. In addition, dual checkpoint blockade strategies incorporating CTLA‐4 inhibition, such as the STRIDE regimen (tremelimumab plus durvalumab), have shown promising efficacy in selected patient populations [19].

More recently, the phase III CheckMate 9DW trial further expanded the first‐line immunotherapy landscape by demonstrating superior efficacy of nivolumab plus ipilimumab compared with lenvatinib or sorafenib in systemic therapy‐naïve unresectable HCC. In the expanded analysis, nivolumab plus ipilimumab achieved an ORR of 36% versus 13% with lenvatinib or sorafenib, including higher complete response rates and durable responses, together with a significant overall survival benefit. These findings support nivolumab plus ipilimumab as an additional first‐line standard‐of‐care option and are expected to be reflected in forthcoming guideline updates [21].

Importantly, most pivotal clinical trials evaluating immune checkpoint inhibitors in HCC have predominantly enrolled patients with preserved liver function, typically Child‐Pugh class A cirrhosis. Consequently, safety and efficacy data in patients with Child‐Pugh B remain limited. Nevertheless, real‐world studies and recent phase II trials suggest that selected patients with Child‐Pugh B7 liver function may still derive benefit from immunotherapy [22, 23].

Despite these advances, response rates remain heterogeneous across first‐line immunotherapy regimens, and a substantial proportion of patients still exhibit either primary or acquired resistance to immune checkpoint inhibition [24]. Several mechanisms may contribute to this limited efficacy, including the presence of an immunologically “cold” tumour microenvironment characterized by low T‐cell infiltration, immunosuppressive stromal components, and metabolic alterations that impair effective anti‐tumour immune responses [12, 25]. In addition, the lack of reliable predictive biomarkers complicates patient selection and limits the ability to identify individuals who are most likely to benefit from checkpoint blockade.

Furthermore, immune‐related adverse events (irAEs) remain an important clinical consideration, particularly in patients with underlying cirrhosis and compromised liver function (Table 1). In patients with HCC, elevations of liver enzymes during immune checkpoint therapy require careful differential diagnosis, as they may reflect immune‐mediated hepatitis, drug‐induced liver injury (DILI), viral hepatitis flare or reactivation, tumour‐ or cirrhosis‐related hepatic deterioration [26]. This distinction is particularly critical in HCC, where most patients present with underlying chronic liver disease and limited hepatic reserves [27]. Recent studies indicate that immune‐related liver injury may occur more frequently and earlier in patients with HCC compared with other solid tumours [28], highlighting the importance of integrating clinical history, viral status, imaging findings, and the temporal pattern of liver test abnormalities when evaluating hepatotoxicity during immunotherapy [29].

**TABLE 1:** Differential characteristics of drug‐induced liver injury and immune‐mediated hepatitis during systemic therapy for HCC.

Feature	Drug‐induced liver injury (DILI)	Immune‐mediated hepatitis
Pathophysiology	Direct or idiosyncratic hepatotoxic effect of the drug (e.g., tyrosine kinase inhibitors such as sorafenib)	Immune‐related adverse event caused by checkpoint inhibitor; induced T‐cell attack on hepatocytes
Typical onset	Usually within days to weeks after initiation of a TKI or other hepatotoxic agent	6–12 weeks after initiation of immunotherapy, but may occur earlier
Autoantibodies	Usually absent	May be present (e.g., ANA, ASMA)
Imaging (CT/MRI)	Often normal; occasionally shows hepatic steatosis	May reveal periportal edema or gallbladder wall thickening
Biopsy findings	Zonal hepatocellular necrosis (often zone 3), eosinophilic infiltration, or microvesicular steatosis	Pan‐lobular hepatitis with prominent CD8^+^ T‐cell infiltration
Primary management	Dose reduction or discontinuation of the causative drug	Systemic corticosteroid therapy (e.g., prednisolone or methylprednisolone)
Response to steroids	Typically absent	Rapid improvement of liver enzymes, usually within 48–72 h
Clinical features	Risk of acute liver failure; assessment according to Hy's Law is recommended	Often accompanied by other immune‐related adverse events such as dermatitis, colitis, or thyroiditis

### 1.2 Therapeutic Considerations in Virus‐Associated HCC

**FIGURE 1:**
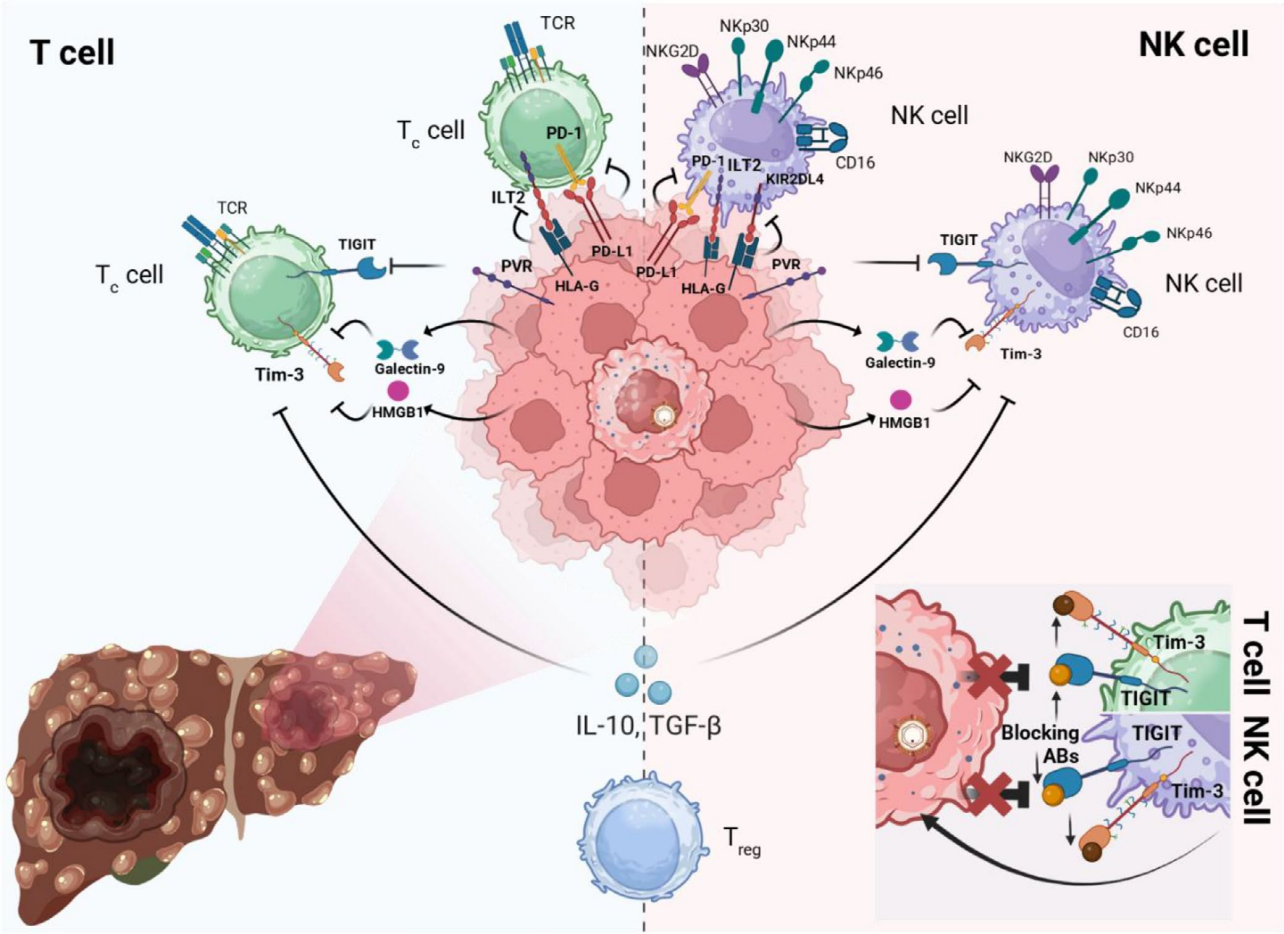
TIGIT and Tim‐3 mediated immune suppression in HCC. Both T cells and NK cells express the inhibitory receptors TIGIT as well as Tim‐3. By binding their respective ligands like soluble Galectin‐9 for Tim‐3 or PVR for TIGIT, they induce intracellular signalling cascades resulting in robust inhibition of T cell and NK cell cytotoxicity. HCC tumour cells also highly express the proteins HLA‐G and PD‐L1, which contribute to immune suppression by binding to ILT2 or PD‐1, respectively.

Chronic infections with HBV or HCV profoundly shape the hepatic immune microenvironment and represent major drivers of HCC development. Persistent viral antigen exposure promotes chronic inflammation and sustained immune activation, which over time leads to immune exhaustion, impaired immune surveillance, and an increased risk of malignant transformation in hepatocytes [30]. In particular, chronic HBV infection modulates anti‐tumour immunity by impairing the functional activity of both NK and virus‐specific T cells, thereby creating an immunosuppressive environment that facilitates tumour development and progression [31, 32, 33] (Figure 1). Within this context, combining antiviral treatment with immunotherapeutic approaches has been proposed as a rational strategy to simultaneously control viral replication and restore effective anti‐tumour immune responses [24, 34]. In clinical practice, effective suppression of viral replication using nucleos(t)ide analogues prior to and during systemic therapy is recommended to minimize the risk of HBV reactivation and to ensure safe administration of immunomodulatory treatments [35, 36, 37]. Nevertheless, integrating antiviral and immune‐based therapies presents several challenges. Immune‐related adverse events (irAEs), particularly hepatic toxicities, require careful clinical management to balance therapeutic efficacy with patient safety [38, 39] (Table 1). Despite growing interest in immune‐based strategies, important knowledge gaps remain. For example, clinical trials evaluating cellular immunotherapies such as CAR T‐ or CAR NK‐cell therapies in HCC have not yet systematically investigated optimal antiviral pre‐treatment or sequencing protocols. Similarly, although immune checkpoint inhibitor–based regimens are currently recommended as first‐line systemic therapy for advanced HCC [17, 40], the optimal integration of antiviral therapy with immunotherapy remains an area of ongoing investigation.

Beyond checkpoint inhibition, emerging clinical and preclinical investigations are exploring the potential of humoral therapies combined with agents or genetically modified immune cells directed against viral proteins in patients with virus‐associated HCC (Table 2) [39, 41]. Preliminary results suggest that these combinations improve overall survival rates and reduce disease progression by simultaneously targeting the viral cause, thus enhancing immune‐mediated tumour control [42].

**TABLE 2:** Current clinical trials investigating cellular immunotherapeutic approaches.

Study ID (ClinicalTrials.gov)	Therapy modality	Target/Mechanism	Phase	Patient population/Indication	Key remarks and outcome
NCT03884751	CAR‐T cells	GPC3‐targeted CAR‐T cells	Phase I	Advanced GPC3‐positive HCC	Evaluation of safety and preliminary efficacy
NCT02723942	CAR‐T cells	GPC3‐targeted	Phase I/II	GPC3‐positive HCC	Early clinical development; safety and feasibility
NCT05003895	CAR‐T cells	GPC3 (hYP7 epitope)	Phase I	GPC3‐positive HCC; dose escalation	Focus on safety, tolerability, and toxicity
NCT02395250	CAR‐T cells	Anti‐GPC3	Phase I	Advanced HCC	Early CAR‐T development; target engagement
NCT04121273	CAR‐T cells	GPC3‐targeted	Phase I	Advanced HCC	Safety, tolerability, and early anti‐tumour activity
NCT06641453	CAR‐T cells	GPC3‐targeted	Phase I/II	GPC3‐positive HCC	Alternative GPC3‐directed CAR‐T construct
NCT05845502	CAR‐NK/cellular therapy	NK cell–based immunotherapy	Terminated	Advanced HCC	NK cell–focused cellular immunotherapy
NCT06968195	Autologous CAR‐T cells	GPC3‐targeted	Phase I	Relapsed or refractory HCC	Focus on treatment‐refractory disease
NCT06251115	CAR‐ / TCR‐based therapy	CAR‐T or TCR‐T cells	NA	Advanced HCC	Broad exploration of cellular immunotherapies
NCT04677088	TCR‐redirected T cells	HBV‐associated HCC antigens	Phase I	HBV‐related HCC	Virus‐specific TCR‐engineered T‐cell approach

### 1.3 Advances in Diagnostics of HCC During Immunotherapy—Liquid Biopsy

In recent years, liquid biopsy (LB) has emerged as a promising diagnostic and monitoring tool in oncology. First introduced by Klaus Pantel and colleagues, the concept refers to the analysis of tumour‐derived material in peripheral blood as a minimally invasive alternative to conventional tissue biopsy [43]. In contrast to traditional diagnostic approaches that rely on imaging and invasive tissue sampling, liquid biopsy enables repeated and dynamic assessment of tumour biology, offering potential advantages for predicting therapeutic response and monitoring tumour progression. For solid tumours, a major advantage lies in its non‐invasive nature and the possibility of longitudinal sampling, which facilitates real‐time disease monitoring while improving patient compliance compared with repeated tissue biopsies [44, 45]. Here, circulating tumour‐derived desoxyribonucleic acids (ctDNA) and circulating tumour cells (CTC) are isolated and analysed [46, 47]. CTCs are shed into the bloodstream from primary or metastatic tumour lesions, whereas ctDNA is released through tumour cell apoptosis, necrosis, or active secretion [48]. Notably, their frequency is very low compared to normal cell‐free DNA and healthy blood cells [49] mandating highly sensitive analytical methods.

Recent studies suggest that liquid biopsy–based biomarkers may provide valuable insights into treatment response during immunotherapy for HCC. In a phase Ib study evaluating sintilimab combined with bevacizumab in intermediate‐stage HCC, prospective analysis of ctDNA levels and the T cell receptor (TCR) repertoire demonstrated that lower ctDNA concentrations and greater TCR diversity were associated with improved tumour response and survival outcomes [50]. Another study, conducted by Mara Egerer and colleagues, found that smaller EV size in treatment responders and decreased vesicle size during atezolizumab plus bevacizumab therapy correlated with prolonged progression‐free survival in advanced HCC [51]. The group of Elena Vargas‐Accarino showed that baseline ctDNA levels could discriminate radiological responders from non‐responders to immune checkpoint inhibitors in advanced HCC [52]. In another study investigating patients treated with nivolumab, specific ctDNA‐detected mutations, including alterations in *PIK3CA* and *BRCA1*, were associated with shorter overall survival, further highlighting the potential of ctDNA profiling for prognostic stratification [53].

## 2 The Immunosuppressive Tumour Microenvironment in HCC

Understanding the tumour microenvironment (TME) is essential for explaining the heterogeneous responses observed with current therapeutic strategies in HCC. As discussed in the context of immunotherapy and biomarker development, treatment responses remain heterogeneous, which is partly explained by the complex cellular and molecular interactions within the TME. This environment consists of tumour cells, stromal components, immune populations, and extracellular matrix structures that collectively promote tumour progression while limiting effective anti‐tumour immunity [25].

Among the immune regulatory mechanisms within the TME, the expression of non‐classical MHC class I molecules such as HLA‐G has gained increasing attention (Figure 1). HLA‐G is aberrantly expressed in a subset of HCCs and exerts potent inhibitory effects through interactions with receptors such as ILT2 and KIR2DL4 on immune cells, thereby impairing effector lymphocyte activity and promoting immune tolerance within the tumour environment [54, 55, 56].

Within this environment, several immunosuppressive cell types actively limit effective anti‐tumour immunity. The key immunosuppressive populations within the TME include tumour‐associated macrophages (TAMs), myeloid‐derived suppressor cells (MDSCs), and regulatory T cells (Tregs), which collectively inhibit the activity of cytotoxic T lymphocytes (CTLs) and natural killer (NK) cells [25, 57, 58].

TAMs are a predominant cell type within the TME and exhibit significant heterogeneity in their distribution and function [59]. These cells often form a spatial barrier in the peripheral stroma of tumours, impeding the infiltration and activation of effector immune cells such as CD8+ T cells [60]. This barrier is a critical factor in promoting tumour immune evasion and progression. TAMs in HCC are associated with the suppression of T cell activity through the expression of inhibitory ligands like PD‐L1, TIM‐3, TIGIT, or HLA‐G and the secretion of immunosuppressive cytokines such as IL‐10 and TGF‐β [61] (Figure 1). These findings highlight the importance of targeting TAMs in therapeutic strategies aimed at modulating the TME to enhance anti‐tumour immunity [61, 62].

MDSCs represent another important immunosuppressive population within the TME [63]. These cells accumulate in HCC lesions and inhibit T‐cell responses through multiple mechanisms, including the production of reactive oxygen species, nitric oxide, and arginase, which impair T‐cell receptor signalling and reduce nutrient availability required for T‐cell proliferation [61, 64]. Additionally, MDSCs can recruit and activate Tregs, further amplifying the immunosuppressive environment [65, 66]. The presence of high levels of MDSCs in the TME correlates with poor prognosis in HCC patients, underscoring the need for therapeutic interventions targeting MDSC activity [67].

Tregs further contribute to immune tolerance within the tumour environment. While these cells normally maintain immune homeostasis and prevent autoimmunity, their accumulation in HCC suppresses effector T‐cell and NK‐cell responses through inhibitory cytokines and checkpoint signalling [68, 69, 70, 71]. Notably, HLA‐G may indirectly promote the induction and stability of Tregs, supporting their persistence within the tumour microenvironment [72, 73].

The immunosuppressive and heterogenous nature of the TME in HCC presents significant challenges for effective immunotherapy. However, recent studies have provided valuable insights into potential therapeutic strategies aimed at modulating the TME to improve the efficacy of immune‐based treatments [74, 75]. For instance, targeting TAMs with reprogramming agents or inhibitors of TAM recruitment, for example, anti‐macrophage colony‐stimulating factor 1 (CSF‐1) antibody, has shown promise in preclinical models [76, 77, 78, 79, 80]. Additionally, therapies that target MDSCs, either through depletion or inhibition of their immunosuppressive functions, using for example, CXCR1/2 or STAT3 inhibitors [81, 82, 83], are actively being explored as potential adjuncts to existing immunotherapies [84, 85]. Moreover, combining immune checkpoint inhibitors with therapies that target the TME, such as TAM or MDSC inhibitors, or directly addressing HLA‐G‐mediated immune suppression holds significant potential for overcoming the immunosuppressive barriers that currently limit the effectiveness of treatments. These combination strategies are under evaluation in clinical trials and could represent a new frontier in HCC management [86, 87].

Beyond the cellular composition of the TME, there is the architectural remodelling of the liver caused by cirrhosis which leads to portal hypertension as well as shunting and thus may compromise the efficacy of CAR T and NK cells. So far, cirrhosis‐induced architectural changes have been identified as a critical but largely uninvestigated barrier to cellular therapy delivery in HCC, with only limited mechanistic evidence currently available [88]. Singular studies show that in patient‐specific cirrhotic livers locoregional particle distribution differs significantly between patients after systemic application and thus mandates locoregional application [89]. Portal vein injections of anti‐GPC3 CAR‐T cells showed improved efficacy in an HCC xenograft mouse model compared to systemic injections [90].

## 3 Advantages in HCC Therapy

### 3.1 Emerging Immune Checkpoints: TIGIT and Tim‐3

Current immune checkpoint inhibitor‐based HCC therapies target the proteins PD‐1/PD‐L1 and CTLA‐4. Notably, recent advances have identified further immune checkpoints such as TIGIT and Tim‐3 as regulators of anti‐tumour immunity [91, 92] and are currently investigated as listed in clinical studies (Table 3). These molecules are expressed on T‐ and NK cells and are frequently upregulated in chronically inflamed or metabolically altered liver environments, contributing to immune exhaustion and impaired anti‐tumour responses [93]. In HCC, both TIGIT and Tim‐3 are frequently upregulated within the tumour microenvironment (TME) (Figure 1), where they contribute to the suppression of anti‐tumour immune responses [94, 95, 96]. Correspondingly, their ligands are also highly expressed on HCC cells and thus contribute to immune suppression, that is, PVR (CD155) and PVRL1 (nectin‐1) for TIGIT [97, 98] as well as galectin‐9 and HMGB1 for Tim‐3 [99, 100, 101], which may presents potential predictive biomarkers.

**TABLE 3:** Current clinical trials targeting TIGIT or Tim‐3 immune checkpoints in HCC.

Study ID (ClinicalTrials.gov)	Checkpoint target	Therapeutic agent	Combination strategy	Phase	Key remarks and outcome
NCT04524871	TIGIT	Tiragolumab	Atezolizumab ± Bevacizumab	Phase II	TIGIT blockade combined with standard ICI backbone
NCT05019900	TIGIT	Ociperlimab (BGB‐A1217)	Tislelizumab	Phase I/II	Early efficacy and immune activation assessment
NCT04639180	TIGIT	Vibostolimab	Pembrolizumab	Phase I	Basket study including HCC cohorts
NCT03680508	Tim‐3	Sabatolimab (MBG453)	Spartalizumab (anti–PD‐1)	Phase I/II	Targeting T‐cell exhaustion in solid tumours
NCT02817633	Tim‐3	TSR‐022	TSR‐042 (anti–PD‐1)	Phase I	Early clinical evaluation of Tim‐3 blockade
NCT04139902	Tim‐3	INCAGN02390	Nivolumab	Phase I	Safety and immune modulation profiling
NCT05787444	TIGIT	EOS‐448	Nivolumab ± Ipilimumab	Phase I	NK cell–biased TIGIT targeting strategy

Functionally, these checkpoint pathways suppress cytotoxic T cell and NK cell activity, thereby facilitating tumour immune escape [102, 103]. Several studies have demonstrated that TIGIT expression is elevated on NK cells in HCC, leading to impaired NK cell cytotoxicity and reduced tumour control [94, 95, 104, 105]. Blocking TIGIT signalling has been shown to restore NK cell function and enhance anti‐tumour responses in experimental models (Figure 1). Similarly, the groups of Ganjalikhani et al. as well as Gardner et al. showed that Tim‐3 upregulation on T cells in HCC contributes to T cell exhaustion within the tumour [106]. Inhibition of Tim‐3 led to the reactivation of exhausted T cells, improved immune responses, and significant tumour regression in preclinical models [80]. The upregulation of TIGIT and Tim‐3 in the HCC microenvironment exacerbates immune evasion, highlighting the need for therapies that can overcome these specific inhibitory pathways. As discussed earlier, blocking these checkpoints can enhance anti‐tumour immunity, and at the same time can cause autoimmunity or other irAEs [107].

### 3.2 Glypican‐3‐Directed Cellular Immunotherapies

**FIGURE 2:**
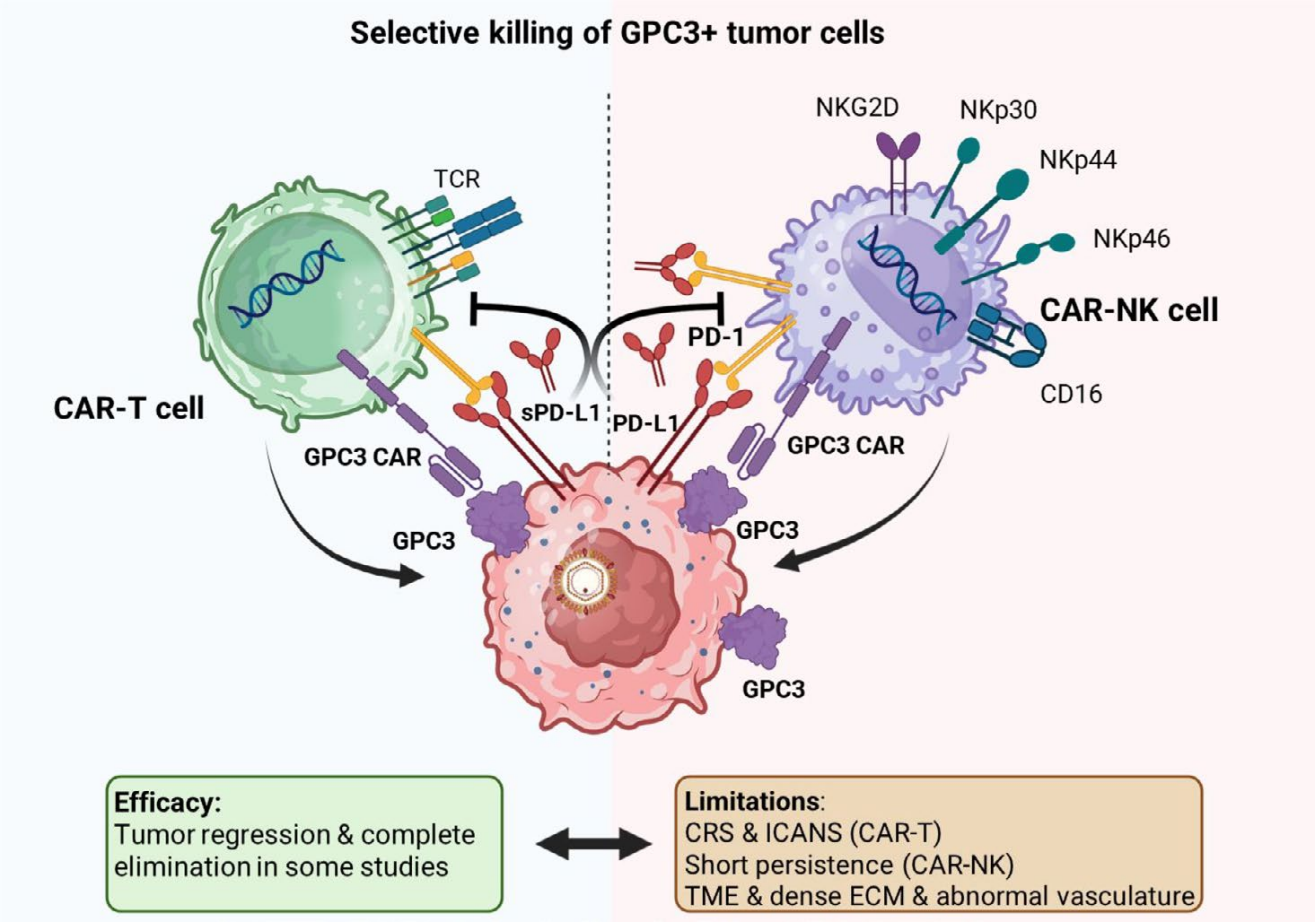
GPC3‐specific CAR T cells and CAR NK cells target and kill GPC3+ HCC tumour cells. T cells and NK cells can be genetically modified to express a CAR specific for the HCC tumour antigen GPC3. Thus, they bind HCC tumour cells and effectively eliminate them through their distinct cytotoxic effector mechanisms, for example, apoptosis. This effect is supported by applying immune checkpoint inhibitors, for example, anti‐PD‐1 or anti‐PD‐L1 antibodies.

Glypican‐3 (GPC3) is an oncofetal protein that is highly expressed in HCC but absent in healthy adult liver tissues. This selective expression pattern makes GPC3 an ideal target for immunotherapeutic strategies. GPC3 is implicated in regulating cell growth and differentiation and contributes to tumour progression through the activation of signalling pathways such as Wnt/β‐catenin, which is known to play a crucial role in HCC oncogenesis [108]. Recent preclinical and clinical studies, as listed in Table 2, have explored the potential of GPC3‐specific chimeric antigen receptor (CAR)‐T and CAR‐NK cells as therapeutic tools for HCC [109, 110, 111] (Figure 2). These studies provided compelling evidence that CAR‐T and CAR‐NK cells engineered to target GPC3 can selectively recognize and kill GPC3‐expressing HCC cells. GPC3‐CAR‐T cells have shown to be generally well‐tolerated and can achieve significant tumour regression, with one study even reporting complete tumour elimination. However, the therapy is not without limitations. Toxicities such as cytokine release syndrome (CRS) and immune cell associated neurotoxicity syndrome (ICANS) present significant safety concerns [112]. Additionally, the TME, characterized by high levels of Tregs, MDSCs, and inhibitory cytokines, displays a significant barrier to CAR‐T cell efficacy [58]. Recent studies further identified TREM2+ tumour‐associated macrophages (TAM) as mediators of resistance to GPC3‐CAR‐T cell therapy, with TREM2 deficiency synergizing with GPC3‐CAR‐T cells to enhance tumour control [113]. Shed GPC3 (sGPC3) hinders CAR‐T cell therapy by competing with cell surface GPC3 for binding, inhibiting cytokine release and cytotoxicity, acting as a dominant negative regulator in HCC [114].

On the other hand, GPC3‐specific CAR‐NK cells have demonstrated potential in early‐phase clinical trials and preclinical studies (Table 2) showing effective antitumor responses with a favourable safety profile and lower risk of severe adverse effects such as graft‐versus‐host disease (GVHD). However, their clinical efficacy can be limited by reduced in vivo persistence and susceptibility to inhibitory signals within the TME, including soluble PD‐L1 (Figure 2) [115, 116, 117]. In this context, combining CAR‐NK cells with the high‐affinity sPD‐L1 variants, like L3C7c‐Fc, can enhance the antitumor activity of CAR‐NK cells [117]. Additional approaches focus on modulating tumour‐intrinsic pathways that contribute to immune evasion. Furthermore, combining Lin28 inhibition with GPC3‐CAR T cell therapy in an HCC mouse model significantly improved anti‐tumour activity, suggesting that targeting Lin28B could enhance the efficacy of immunotherapies against HCC by reducing IDO1 and PD‐L1 expression [118]. These findings are particularly relevant in metabolically driven HCC, such as MASLD/MASH‐associated tumours, where metabolic reprogramming and accumulation of immunosuppressive metabolites can contribute to functional exhaustion of T‐ and NK‐cells and promote an immunologically “cold” tumour microenvironment [119, 120].

For example, the RNA‐binding protein Lin28B has been implicated in the regulation of immune suppressive pathways in HCC. Lin28B overexpression increases IDO1 expression and kynurenine production, promoting immune escape and PD‐L1 expression. Inhibition of Lin28B reduces these effects and enhances the cytotoxic activity of GPC3‐targeted CAR‐T and CAR‐NK cells in preclinical models [118].

Interestingly, GPC3‐specific CAR constructs have been adapted for macrophages, exploiting their intrinsic ability to infiltrate solid tumours and mediate phagocytosis. Similar to CAR‐T cells, CAR macrophages contain intracellular activation signalling domains that trigger macrophage‐mediated tumour cell engulfment [121]. Thus, in experimental models, GPC3‐targeted CAR macrophages demonstrated antigen‐specific phagocytosis and tumour killing in both two‐ and three‐dimensional HCC models [122]. Targeting tumour‐induced stress proteins with CAR macrophages further resulted in complete tumour regression and 100% survival in orthotopic HCC models and showed synergy with anti‐PD‐L1 therapy [123].

These findings are particularly relevant in metabolically driven HCC, such as MASLD/MASH‐associated tumours, where metabolic reprogramming and accumulation of immunosuppressive metabolites can contribute to functional exhaustion of T‐ and NK‐ cells and promote an immunologically “cold” tumour microenvironment [119, 120]. Here, the combination of antigen‐redirecting platforms with bispecific antibodies represents promising strategies to further tackle these limitations and overcome resistance [10, 124].

### 3.3 GPC3‐Targeted Bispecific Antibodies

**FIGURE 3:**
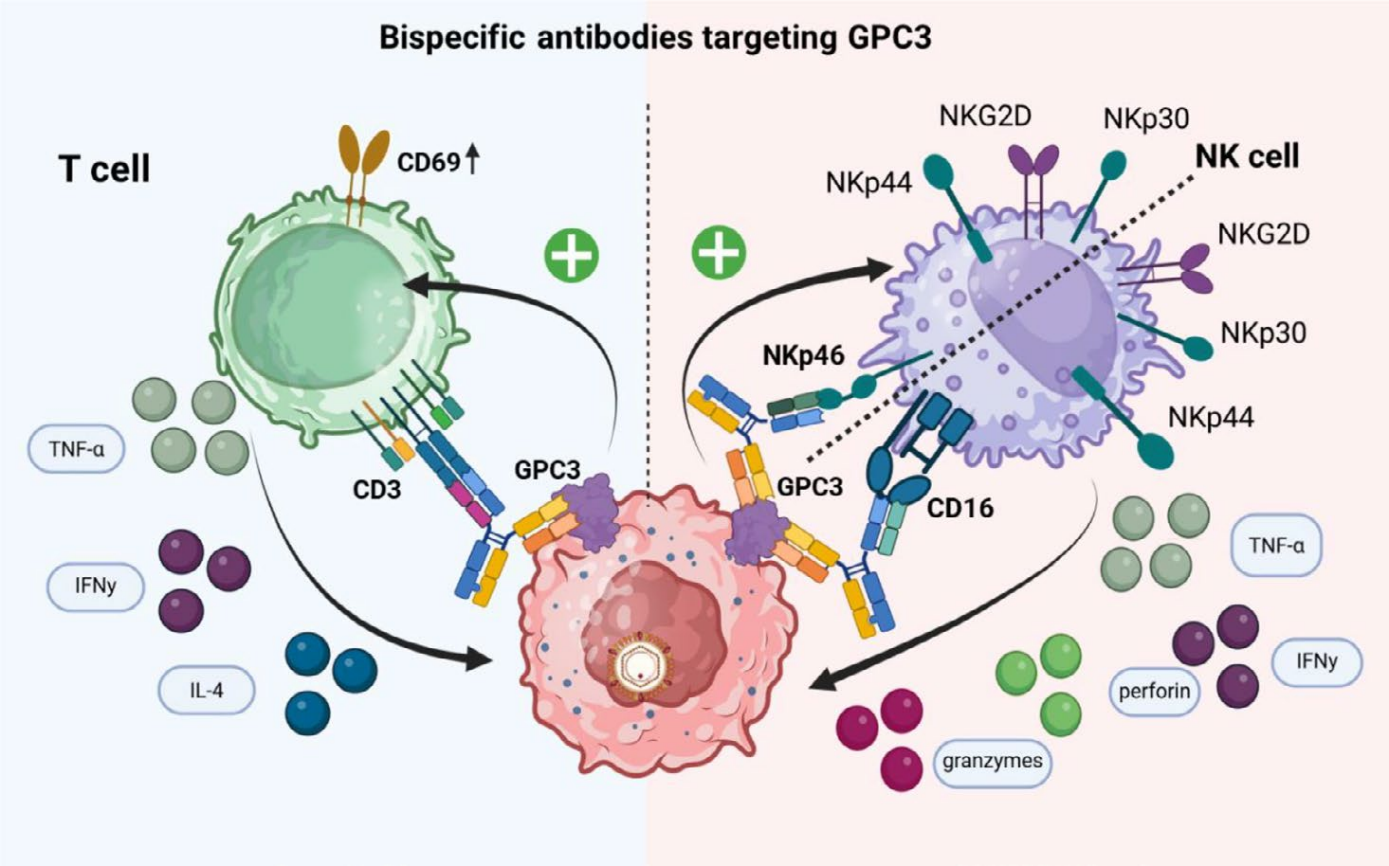
Bispecific antibodies bridge GPC3+ HCC tumour cells and T cells as well as NK cells. Bispecific antibodies target simultaneously GPC3 on HCC tumour cells and CD3 on T cells or KIRs as well as CD16 on NK cells, respectively. Thus, bringing effector and target cells in close proximity, cytotoxicity is induced and the tumour cells are effectively destroyed.

Beyond CAR‐based strategies, bispecific antibodies (BsAbs) targeting GPC3 have emerged as a promising immunotherapeutic platform for HCC [125] (Figure 3). Several preclinical studies have demonstrated that GPC3 × CD3 constructs can potently redirect T cells against GPC3‐positive tumour cells. Early BiTE formats [126, 127] and IgG‐based tetravalent antibodies [128, 129] induced strong T cell activation, cytokine release (TNF‐α, IFN‐γ, IL‐4), and efficient cytotoxicity, with in vivo studies confirming tumour growth suppression. Importantly, some of these agents showed efficacy at very low concentrations (as low as 0.01 ng/mL) and synergized with chemotherapeutics such as irinotecan. More advanced designs, including h8B‐BsAb [128] with improved stability and productivity, achieved sustained tumour regression in xenograft models. Parallel efforts have developed BsAbs to mobilize innate immunity. Constructs engaging CD16A or NKp46 [130, 131, 132] effectively activated NK cells (Figure 3), enhanced antibody‐dependent cellular cytotoxicity (ADCC), and demonstrated tumour inhibition, with one candidate (CYT‐303) showing safety in cynomolgus monkeys and another (MA4‐hFc‐CD16AM19) exhibiting synergy with sorafenib in Huh7 xenografts. Novel strategies also address antigen heterogeneity, such as the use of GPC3 CAR T cells combined with B7H3/CD3 BiTEs [133], which enhanced T cell activity in dual‐positive tumours. In addition, a GPC3/CD47 BsAb [134] was shown to boost innate effector functions by macrophages and neutrophils, prolong serum half‐life, and outperform the combination of anti‐GPC3 and anti‐CD47 monoclonal antibodies in xenograft models, thereby highlighting the potential of dual checkpoint and tumour antigen targeting. Collectively, these findings demonstrate that GPC3‐directed BsAbs can recruit both adaptive and innate immune compartments, achieve potent anti‐tumour activity, and synergize existing therapies, while early safety assessments suggest a manageable toxicity profile (Figure 3). Although most candidates remain at the preclinical stage, ERY974 represents the first GPC3 × CD3 BsAb tested clinically (NCT02748837), where it showed tolerability and early signs of activity in a phase I trial [125, 135]. Together, these developments position GPC3‐targeted BsAbs as a versatile, dose‐controllable, and potentially safer alternative to CAR‐modified cell therapies in HCC.

## 4 The Role of NK Cells in HCC Therapy

### 4.1 Tumour Surveillance and Therapeutic Resistance

NK cells are essential components of the innate immune system, with a critical role in the surveillance and elimination of malignant cells. These cells can recognize and kill tumour cells without prior sensitization, primarily through direct cytotoxicity and the production of cytokines such as interferon‐gamma (IFN‐γ), which further enhances the adaptive immune response. NK cell activity is modulated by a balance of activating and inhibitory signals, making their function tightly regulated within the TME [136]. In the context of HCC, the function of NK cells is often impaired, leading to reduced tumour surveillance and increased tumour progression. The dysfunction has been shown to be mediated by critical immune checkpoints such as PD‐1, TIGIT, CD112R, CD96, IL‐1R8, and TIM‐3 within the TME [137]. In addition, metabolically driven alterations within the TME can further impair NK cell cytotoxicity and contribute to functional exhaustion of innate immune responses. The overexpression of these inhibitory molecules on NK cells impairs their cytotoxicity, allowing tumour cells to evade immune destruction [95, 137]. Blocking the inhibitory molecules has been shown to restore NK cell activity, leading to increased tumour cell lysis and potentially controlling tumour growth [105]. A recent study on the protein Sialic acid‐binding Ig‐like lectin 9 (Siglec‐9) demonstrated that its interaction with its ligand on tumour cells leads to a significant inhibition of NK cell activity [138]. This interaction effectively inactivates the NK cells, reducing their ability to kill HCC cells and contributing to tumour immune evasion. The impaired function of NK cells in HCC represents a significant barrier to the efficacy of immunotherapies that relieve the activation of these cells. In other malignancies, such as hematologic cancers, NK cell‐based therapies have shown considerable promise [139]. However, the unique and complex interactions within the HCC TME present challenges that necessitate innovative therapeutic approaches to restore NK cell activity. The TME in HCC is particularly hostile to NK cells due to the presence of various immunosuppressive molecules, as mentioned, and cell types, including TAMs, MDSCs, and Tregs, which further inhibit NK cell function. To overcome this complex tumour protective network, therapeutic strategies to enhance NK cell function in HCC involve multiple approaches, including the blockade of inhibitory receptors, such as NKG2A, cytokine therapy to boost NK cell activity, for example, IL‐15, and combination therapies that target multiple aspects of the immunosuppressive TME, need to be further elucidated [140, 141, 142, 143].

Of note, regarding general treatment access barriers in HCC‐endemic low‐ and middle‐income countries off‐the‐shelf CAR‐NK cells have received strong conceptual support as scalable alternatives for CAR‐T cells, but for HCC evidence remains largely preclinical. The production of NK cell‐based off‐the‐shelf therapeutics can be obtained from unrelated healthy blood donors and pluripotent stem cells. The group of Li et al. introduces an ex vivo feeder‐free culture method to generate allogeneic CAR‐NK T cells from haematopoietic stem cells as a scalable alternative to autologous CAR‐T therapy [144]. Boyd and colleagues describe iPSC‐derived CAR‐NK cells targeting TAG72 in ovarian cancer [145], demonstrating potent in vitro cytotoxicity and scalable manufacturing with reduced costs, and Zuccolotto et al. present dual CAR‐NK92 cells for prostate cancer, showing specific cytotoxicity and cost advantages over CAR‐T [146].

### 4.2 NK Cells and Their Role in Supporting Oncolytic Viral Therapy

**FIGURE 4:**
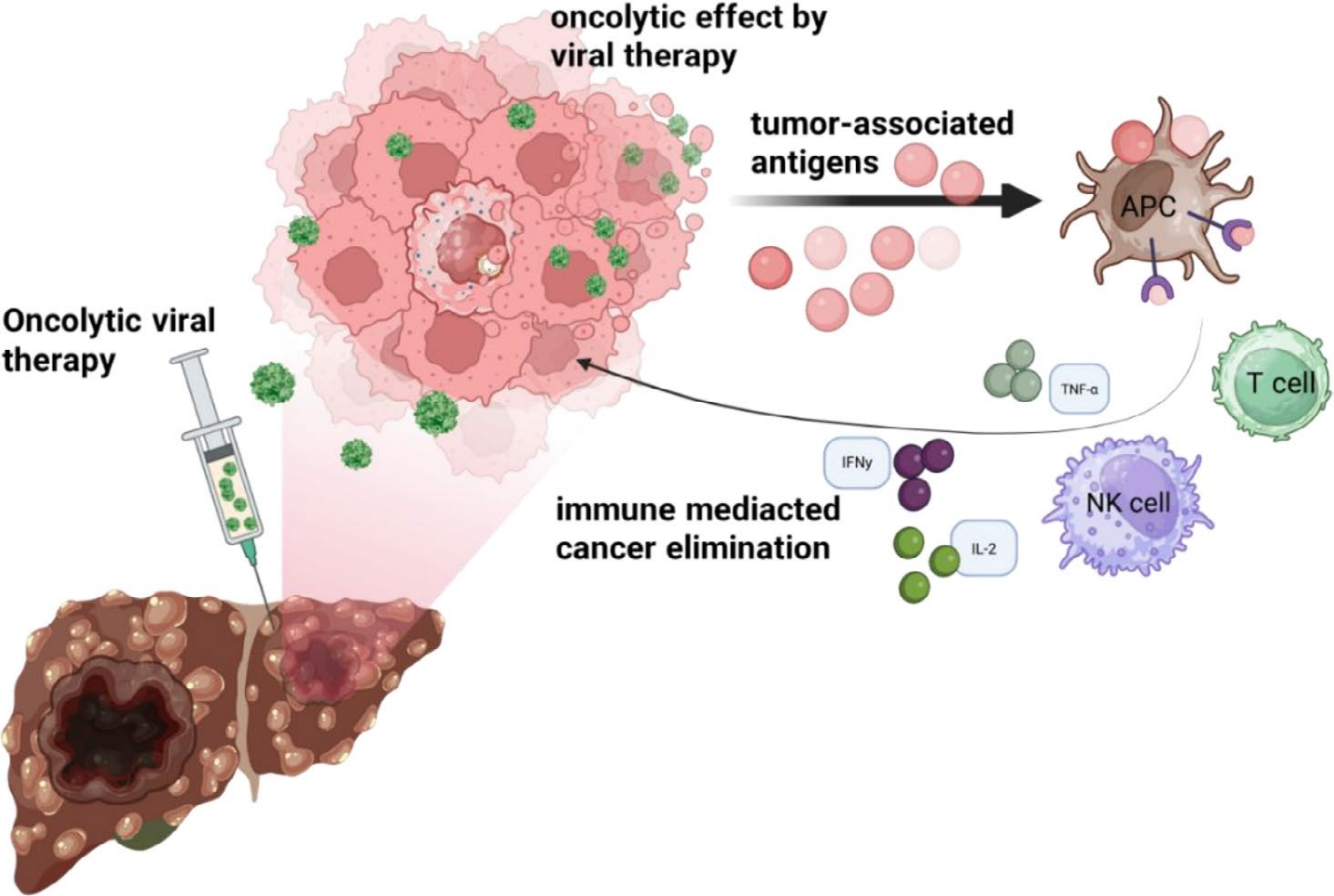
Oncolytic viruses specifically target and destroy tumour cells. Genetically modified viruses capable of tumour antigen dependent binding and infection of tumour cells lead to controlled elimination of tumour tissues. Thus, more tumour antigens are released into the nearby extracellular space and there taken up by APCs, further enhancing the adaptive anti‐tumour immune response.

Oncolytic viruses are genetically engineered to selectively infect and lyse tumour cells while sparing normal tissues (Figure 4). Beyond their direct cytolytic effects, these viruses can stimulate systemic anti‐tumour immune responses by releasing tumour‐associated antigens and danger‐associated molecular patterns during tumour cell lysis. These signals promote the recruitment and activation of antigen‐presenting cells such as dendritic cells, which subsequently prime tumour‐specific T cells and contribute to the conversion of immunologically “cold” tumours into inflamed, immune‐reactive “hot” tumours [147] (Figure 4). The combined application of oncolytic viruses and NK cells showed enhanced efficacy in HCC treatment by contributing significantly to antitumor immunity by directly lysing tumour cells and enhancing the activation of dendritic cells and T cells [148].

However, the study also highlighted a potential drawback: NK cells might eliminate the oncolytic viruses‐infected tumour cells too early, limiting viral replication and reducing the overall efficacy of the therapy. NK cells, through their cytotoxic activity, help to eliminate virus‐infected tumour cells, thereby amplifying the oncolytic effects of the therapy. This dual mechanism of action, direct oncolysis by the virus and immune‐mediated tumour clearance by NK cells, offers a potent anti‐cancer strategy [148]. However, the early elimination of oncolytic viruses by NK cells might prevent the virus from establishing an infection that is sufficient to induce a robust immune response. This finding suggests that a careful balance must be struck between promoting NK cell activity and allowing the virus to persist long enough to trigger a systemic anti‐tumour response. Translating these preclinical observations into clinical practice, multiple oncolytic virus–based therapeutic strategies have been evaluated in patients with hepatocellular carcinoma, either alone or in combination with immunomodulatory agents. A summary of ongoing and completed clinical trials investigating oncolytic virotherapy in HCC is provided in Table 4.

**TABLE 4:** Current clinical trials investigating oncolytic virus‐based therapies in HCC.

Study ID	Oncolytic virus	Virus type/Payload	Combination strategy	Phase	Key remarks and outcome
NCT02562755	Pexa‐Vec (JX‐594)	Vaccinia virus (GM‐CSF)	Sorafenib	Phase III	Failed OS endpoint despite strong immune activation
NCT01387555	Pexa‐Vec	Vaccinia virus (GM‐CSF)	Monotherapy	Phase II	Demonstrated intratumoral replication and immune priming
NCT03206073	Pexa‐Vec	Vaccinia virus (GM‐CSF)	Nivolumab	Phase I/II	Synergistic immune activation with checkpoint blockade
NCT03921021	T‐VEC	HSV‐1	Pembrolizumab	Phase I	Translation of HSV‐based oncolysis to HCC
NCT05076760	VG161	Adenovirus (IL‐12, IL‐15, PD‐L1 scFv)	Monotherapy	Phase I	Multi‐armed vector targeting the immunosuppressive TME
NCT04612504	LOAd703	Adenovirus (CD40L, 4‐1BBL)	± Checkpoint inhibitors	Phase I/II	Potent activation of APCs, T cells, and NK cells

### 4.3 Enhancing ADCC Through Cytokine Activation of NK Cells

Antibody‐dependent cellular cytotoxicity (ADCC) is a crucial immune mechanism in which NK cells recognize and kill tumour cells that have been bound by specific antibodies. This process is facilitated by the interaction between the Fc receptors on NK cells and the Fc region of antibodies that are bound to tumour‐associated antigens. ADCC plays a significant role in the effectiveness of antibody‐based therapies, such as those using monoclonal antibodies (mAbs) like cetuximab, which target epidermal growth factor receptor (EGFR) on cancer cells [149]. It has been demonstrated that cytokines such as interleukin‐2 (IL‐2) and interleukin‐15 (IL‐15) can significantly boost the cytotoxic activity of NK cells. When combined with cetuximab, the cytokines enhanced the ADCC effect, leading to more effective tumour cell killing by a synergistic effect increasing tumour cell lysis [142]. This approach could be particularly beneficial for patients with impaired NK cell functionality, potentially leading to improved clinical outcomes. However, the use of cytokines like IL‐2 and IL‐15 is often limited by severe side effects, for example, CRS. To harness the full potential of cytokine‐mediated enhancement of ADCC, it is crucial to optimize dosing and delivery methods. Lower doses or localized delivery of cytokines might reduce the risk of CRS while still providing the desired immune‐boosting effects. Recent advances in cytokine engineering, such as the development of IL‐2 variants with reduced binding to regulatory T cells, can offer safer options for enhancing ADCC. Combining cytokine activation with other immunotherapeutic strategies, such as immune checkpoint inhibitors or CAR‐T cells, may provide a synergistic effect [150].

## 5 Conclusion and Future Perspectives

Humoral immunotherapy has emerged as a key strategy in the treatment of hepatocellular carcinoma (HCC), addressing the limited efficacy of conventional therapies through targeted modulation of the immune response [17, 25, 39, 41]. Advances in understanding the immune landscape of HCC have highlighted the pivotal role of the tumour microenvironment in promoting immune escape, particularly through dysfunctional NK cell activity and altered stromal signalling [57, 58, 136, 137, 138]. Therapeutic approaches targeting glypican‐3 (GPC3), immune checkpoint inhibition, and oncolytic viruses have demonstrated clinical potential, especially when used in combination to overcome the immunosuppressive tumour milieu [18, 19, 147, 148]. Increasing evidence also supports the integration of cellular immunotherapies, including NK cell‐based strategies, to restore effective tumour surveillance [116, 139]. Future therapeutic development in HCC will increasingly depend on biomarker‐guided patient stratification [25, 42]. While PD‐L1 expression has been explored as a predictive marker, its clinical value remains limited. Emerging multi‐omic approaches integrating genomic, transcriptomic, and proteomic data may enable improved patient stratification [9, 10, 50]. In addition, liquid biopsy strategies such as circulating tumour DNA (ctDNA) and extracellular vesicles (EVs) offer promising tools for real‐time monitoring of tumour dynamics and treatment responses [50, 52].

The molecular landscape of HCC is also shaped by disease aetiology; for instance, HBV‐associated HCC may harbour viral‐derived neoantigens, whereas non‐viral aetiologies such as alcohol‐related liver disease or MASH display distinct inflammatory and metabolic signatures. These differences highlight the need for aetiology‐specific biomarker strategies to guide future immunotherapeutic development [2, 119]. In addition, emerging evidence suggests that the gut–liver axis and the intestinal microbiome influence immune responses in HCC. Increased intestinal permeability in cirrhotic patients can promote bacterial translocation and activation of hepatic Toll‐like receptor pathways, particularly TLR4, contributing to an immunosuppressive tumour microenvironment. Moreover, specific microbial signatures have been associated with differential responses to immune checkpoint inhibitors [151]. For instance, the presence of bacterial taxa such as *Akkermansia muciniphila* has been linked to improved immunotherapy responses across cancer cohorts and was recently shown to complement PD‐1 efficacy in MAFLD‐related HCC models [152, 153]. These observations suggest that microbiome‐targeted interventions may represent an additional strategy to enhance the efficacy of immune‐based therapies.

As multi‐omics profiling continues to deepen our understanding of tumour–immune interactions, future therapies will likely shift toward personalized, cell‐directed immunotherapeutic strategies tailored to individual immune and tumour phenotypes, thereby maximizing efficacy while minimizing systemic toxicity.

## Funding

The German Research Foundation (DFG) funded the study with a grant to J.K. (KA 5390/2‐1) and to W.D. (DA 2138/3‐1). All funding sources supporting the work are acknowledged, and authors have nothing to disclose.

## Conflicts of Interest

The authors declare no conflicts of interest. All co‐authors have seen and agree with the contents of the manuscript.

## Supporting information


**Table S1:** Selected phase III trials of immune‐based systemic therapies in advanced HCC (BCLC stage C).

## Data Availability

Data sharing not applicable to this article as no datasets were generated or analysed during the current study.
