# Supplementary material for: Emerging Immune‐Based Therapeutic Strategies in Hepatocellular Carcinoma
Source: Liver Int. 2026 Jul 1;46(8):e70773. doi: 10.1111/liv.70773 (PMC13323950; doi:10.1111/liv.70773)
Supplement: Supplementary file 1 — Table S1: Selected phase III trials of immune‐based systemic therapies in advanced HCC (BCLC stage C). [file LIV-46-0-s001.docx]

**Supplementary table 1: Selected phase III trials of immune-based systemic therapies in advanced HCC (BCLC stage C)**

| **Trial** | **Therapy regimes** | **Indication (BCLC stage)** | **Outcome (median overall survival)** |
| --- | --- | --- | --- |
| IMbrave150 | Atezolizumab + Bevacizumab | Stage C / Unresectable HCC | 19.2 months |
| HIMALAYA | Tremelimumab + Durvalumab (STRIDE regimen) | Stage C / Unresectable HCC | 16.4 months |
| CARES-310 | Camrelizumab + Rivoceranib | Stage C HCC | 23.8 months |
| CheckMate 9DW | Nivolumab + Ipilimumab | First-line Stage C HCC | 23.7 months |
